# How Does the Remaining Single Kidney Cope After Contralateral Nephrectomy of the Kidney Donor? A Single-Center Cohort Study

**DOI:** 10.7759/cureus.11491

**Published:** 2020-11-15

**Authors:** Abdulrahman Altheaby, Nouf Alharbi, Alaa Alzamil, Elham Alzahrani, Abeer M Alshaia, Basayl Aldowsary, Ghaleb Aboalsamah, Mahfooz Farooqui, Khaled Bin Saad, Ziad Arabi

**Affiliations:** 1 Organ Transplant Center and Hepatobiliary Sciences Department, King Saud bin Abdulaziz University for Health Sciences, King Abdulaziz Medical City, Riyadh, SAU; 2 Medicine, College of Medicine, King Saud bin Abdulaziz University for Health Sciences, Riyadh, SAU; 3 Nephrology, King Saud bin Abdulaziz University for Health Sciences, King Abdulaziz Medical City, Riyadh, SAU; 4 Division of Adult Transplant Nephrology, Department of Organ Transplant Center, King Abulaziz Medical City, Riyadh, SAU

**Keywords:** kidney donor, transplant, unilateral nephrectomy, renal functions, egfr

## Abstract

Introduction

Immediately after kidney donation, the remaining kidney will undergo hyperfiltration and work at a higher level to compensate for the other kidney's loss. It is estimated that 70% of the baseline renal function before the donation is recovered post-donation. However, factors that determine the post-donation renal compensation are not well understood.

Methods

We conducted a retrospective study of 190 consecutive kidney donors who completed a one-year follow-up in order to predict the factors affecting the function of the remaining kidney post-contralateral nephrectomy.

Results

We enrolled 190 living kidney donors who had completed at least one year of follow-up after nephrectomy. Among the participants, 149 (78.4%) were males and 41 (21.6%) were females. The mean age of the participants was 31.33 ±7.9 years and the mean body mass index (BMI) was 25.6 ±3.9 kg/m^2^. Before kidney donation, the mean estimated glomerular filtration rate (eGFR) and serum creatinine were 114.31 ±15.94 ml/min/1.73 m^2^ and 71.60 ±10.62 mmol/min, respectively. At the one-year follow-up, the mean eGFR was 77.97 ±14.44 ml/min/1.73 m^2^ and serum creatinine was 100.84 ±20.15 mmol/min. The female gender [odds ratio (OR): 20.6, 95% CI: 3.9-107.7, p: <0.001] and having a higher baseline eGFR (OR: 8.8, 95% CI: 1.6-45.8, p = 0.01) were found to be significant predictors of having a better eGFR at one year post-nephrectomy.

Conclusions

Female gender and pre-donation low serum creatinine and high eGFR were the significant predictors of better kidney function at one year post-contralateral nephrectomy. However, further studies with longer follow-up durations are needed to better assess the factors that could predict renal compensation and the renal compensation rate's suitability as a prognostic measure for long-term renal outcomes.

## Introduction

Living-donor kidney transplantation is generally the treatment of choice for patients with end-stage renal disease (ESRD) compared to cadaveric-donor transplantation [1,2]. There is a compelling shortage of live donors in parallel to the growth of demand due to the rising prevalence and incidence of chronic renal disease associated with kidney donation. Before kidney donation, donors undergo extensive medical evaluations to be declared fit for kidney donation, which has further reduced the number of eligible donors [3-6].

Given the favorable outcomes of living kidney donation and the shortage of organs to transplant, individuals with potentially unfavorable demographic and clinical characteristics are increasingly being permitted to donate kidneys. While this trend has successfully expanded the live donor pool, it has raised concerns regarding the prevailing acceptance criteria for donors [4,5]. The perioperative mortality and morbidity related to unilateral nephrectomy are up to 0.03% and 10%, respectively [6,7]. However, several studies have proven that unilateral nephrectomy usually does not lead to any clinically significant short- or long-term renal impairment in the remaining kidney [3,8-10].

The changes in the remaining kidney function of the donor have been documented in various studies. For instance, the glomerular filtration rate (GFR) decreases immediately after unilateral nephrectomy. At one year after donation, the remaining kidney manages to contribute on its own up to 70% of the two kidneys' function [11-13]. Similarly, hemodynamic changes in the remnant kidney have been reported immediately after nephrectomy, such as vasodilation and increased renal plasma flow (RPF) [11,14]. In addition to hypertrophy of glomerulus, these hemodynamic changes will boost the glomerular filtration of the remaining kidney to approximately 40% without a concomitant increase in the glomerular capillary pressure [15,16]. On the other hand, a few studies have reported renal damage among kidney donors to the extent that a few cases warranted renal replacement therapy among the donors themselves [17,18].

Despite the considerable demand for living kidney donors and the extensive investigations they go through to be accepted for kidney donation, there is a paucity of studies that evaluate the changes in the remaining kidney after contralateral nephrectomy among donors. Consequently, there is insufficient data about the factors that can predict the ability of the remaining kidney to compensate for functional renal loss post-contralateral nephrectomy.

In light of the above-mentioned scarcity of data, the present study was conducted to assess the changes in renal function after contralateral nephrectomy and to identify the factors that can predict the degree of renal-function compensation after nephrectomy among kidney donors in our transplant center.

## Materials and methods

This was a retrospective observational study of a cohort of renal donors consecutively undergoing nephrectomy for renal donation between January 2016 to June 2019 at the King Abdulaziz Medical City in Riyadh, Saudi Arabia. We enrolled all living kidney donors who had completed at least one year of follow-up after nephrectomy. Donors who did not undergo a follow-up in the first year following nephrectomy were excluded.

Data were extracted from electronic medical records (BESTCare; ezCaretech, Seoul, Korea) at the hospital. It included patient information about age, gender, body mass index (BMI), smoking status, obesity levels, and renal function parameters such as estimated glomerular filtration rate (eGFR) and serum creatinine, which were collected pre-nephrectomy and at regular intervals post-nephrectomy.

Renal function was assessed based on serum creatinine and eGFR, which was calculated using the modified Modification of Diet in Renal Disease (MDRD) equations, which is a creatinine-based estimate of renal function. The creatinine value and eGFR closest to the donation date were taken as the pre-donation baseline level. We evaluated the change of renal function by looking at serum creatinine and eGFR immediately after nephrectomy, and at one week, one month, six months, and one year of donation.

Statistical analysis

Statistical analyses were performed using IBM SPSS Statistics for Windows version 24 (IBM, Armonk, NY). Continuous variables were presented as mean ±SD and were compared using paired t-test, Student’s t-test, or general linear model for repeated measures as appropriate. Categorical variables were presented as percentages and analyzed using the chi-square test. Linear regression assumptions were checked, and then a model was fitted to identify factors associated with independent study variables. Logistic regression was used to identify significant predictors of eGFR of more than 70 after one year. All the tests were two-sided, and p-values of <0.05 were considered statistically significant.

## Results

Demographic profile of the participants

A total of 190 donors were enrolled in our study. Among them, 149 (78.4%) were males and 41 (21.6%) were females. The majority of them (64.0%) had blood group O. The mean age of the donors was 31.33 ±7.9 years with a mean BMI of 25.6 ±3.9 kg/m^2^; 27 (14.2%) donors were considered obese with a BMI of >30 but <35 as we do not accept people with a BMI of >35 to be a kidney donor as per our protocol. The mean fasting blood sugar level was 5.12 ±0.5 mmol/L. Only two participants were known hypertensives, and they were on a single antihypertensive agent. Seventy-seven (40.5%) donors were smokers. The mean size of the right kidney was 10.5 ±0.8 cm and that of the left kidney was 10.8 ±0.8 cm; 163 (86.3%) donors underwent left nephrectomy and 26 (13.2%) had right nephrectomy (Table 1).

**Table 1:** Baseline demographic data of the donors FBS: fasting blood sugar; HbA1c: glycated hemoglobin; BMI: body mass index; HTN: hypertension; SD: standard deviation

Variables		Values
		Mean ±SD
Age, years		31.3 ±7.9
FBS, mmol/L		5.1 ±0.5
HbA1c, %		5.2 ±0.3
Right kidney size, cm		10.5 ±0.8
Left kidney size, cm		10.8 ±0.8
BMI, kg/m^2^		25.6 ±3.9
		N (%)
Gender	Male	149 (78.4%)
	Female	41 (21.6%)
HTN		2 (1.1%)
Smoker		77 (40.5%)
Blood group	A	31 (16.4%)
	AB	1 (0.5%)
	B	36 (19.1%)
	O	122 (64.0%)
	Rh-positive	179 (94.2%)
	Rh-negative	11 (5.8%)
BMI	Underweight	12 (6.3%)
	Normal	66 (34.7%)
	Overweight	85 (44.7%)
	Obese	27 (14.2%)
Nephrectomy	Left	164 (86.3%)
	Right	26 (13.2%)
Number of arteries in the left kidney	one	162 (85.3%)
	multiple	27 (14.7%)
Number of arteries in the right kidney	one	153 (81.0%)
	multiple	36 (19.0%)
Cyst	one	2 (1%)
	Multiple	2 (1%)

Changes in renal function after contralateral nephrectomy

The eGFR was calculated and creatinine levels were measured pre-nephrectomy and at various times post-nephrectomy. At the baseline, the mean eGFR and mean creatinine measurements were 114.3 ±15.9 ml/min/1.73 m^2^ and 71.5 ±10.6 mmol/min, respectively. As shown in Table 2, a repeated-measures analysis of variance (ANOVA) with a Greenhouse-Geisser correction showed that mean eGFR varied significantly between time points (p: <0.001). Post-hoc tests using the Bonferroni correction revealed a significant eGFR drop of -37.1 (95% CI: -40.9 to -33.2), (p: <0.001) on the first day after surgery. Nevertheless, the difference between the first-day and one-year eGFR was not significant (0.735, 95% CI: -0.8-2.3, p = 1). Similarly, creatinine increased by 32.0 (95% CI: 28.8-35.2, p: <0.001) on the first day, but reduced after one year by -2.8 (95% CI: -5.4 to -0.1, p = 0.030). Additionally, we found a significant rise in BMI after six months by 0.39 (95% CI: 0.20-0.58, p: <0.001). There was no significant change in systolic BP at 12-month follow-up, but diastolic BP and mean arterial pressure (MAP) were significantly increased on the first day by 2.8 (95% CI: 1.3-4.3, p: <0.001) and 1.8 (95% CI: 0.5-3.1, p = 0.007) respectively and remained at the same level during the follow-up period (Table 2).

**Table 2:** Donor eGFR, creatinine, BP, and BMI values pre- and post-kidney donation eGFR: estimated glomerular filtration rate; BP: blood pressure; MAP: mean arterial pressure; BMI: body mass index; SD: standard deviation

Variables	Preoperation, mean ±SD	On day one, mean ±SD	At one week, mean ±SD	At one month, mean ±SD	At six months, mean ±SD	At one year, mean ±SD	P-value
Creatinine, mmol/min	71.5 ±10.6	103.6 ±20.2	101.4 ±21.9	104.9 ±20.0	101.9 ±18.9	100.8 ±20.1	<0.001
eGFR, ml/min/1.73 m^2^	114.3 ±15.9	77.2 ±15.0	77.6 ±14.8	74.3 ±12.8	77.2 ±13.0	77.9 ±14.4	<0.001
Systolic BP, mmHg	121.9 ±11.9	121.8 ±11.8			123.4 ±10.3	123.0 ±10.0	0.143
Diastolic BP, mmHg	69.8 ±10.2	72.7 ±8.6			71.6 ±8.1	71.5 ±8.6	0.002
MAP, mmHg	87.2 ±9.3	89.0 ±8.5			88.9 ±7.6	88.6 ±7.7	0.016
BMI, kg/m^2^	25.6 ±3.9				26.0 ±4.0		<0.001

Variables predicting renal function after contralateral nephrectomy

We entered age, baseline eGFR, baseline BMI, MAP, kidney size, gender, smoking status, HTN, side of nephrectomy, and the number of renal arteries into linear regression analysis as independent variables. After controlling for these risk factors, we found that a higher baseline eGFR, female gender, and a bigger contralateral kidney size were associated with a higher eGFR after one year. Conversely, younger age and higher baseline BMI were associated with a lower eGFR after one year (Table 3).

**Figure 1:**
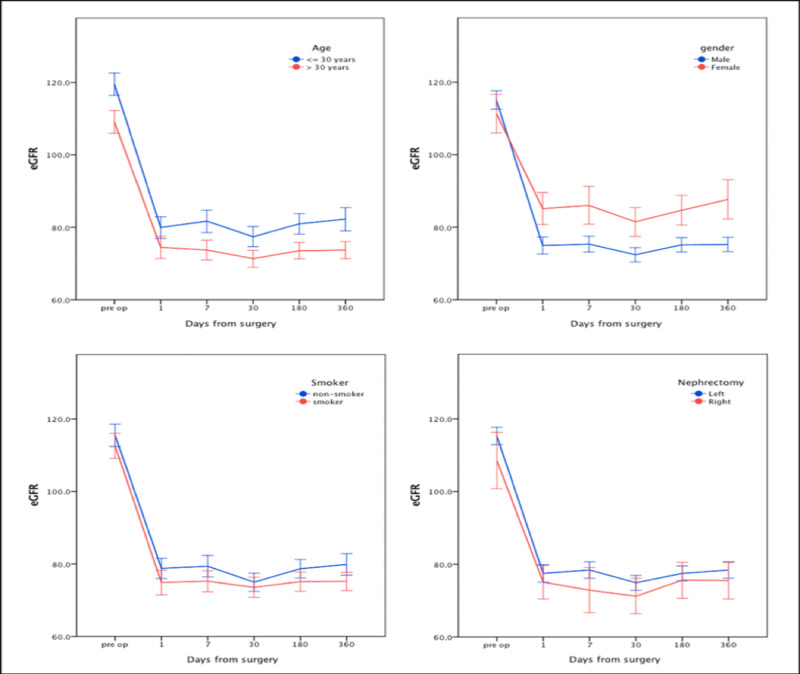
Time course changes in eGFR in the experimental groups (error bars represent 95% CI) eGFR: estimated glomerular filtration rate

**Figure 2:**
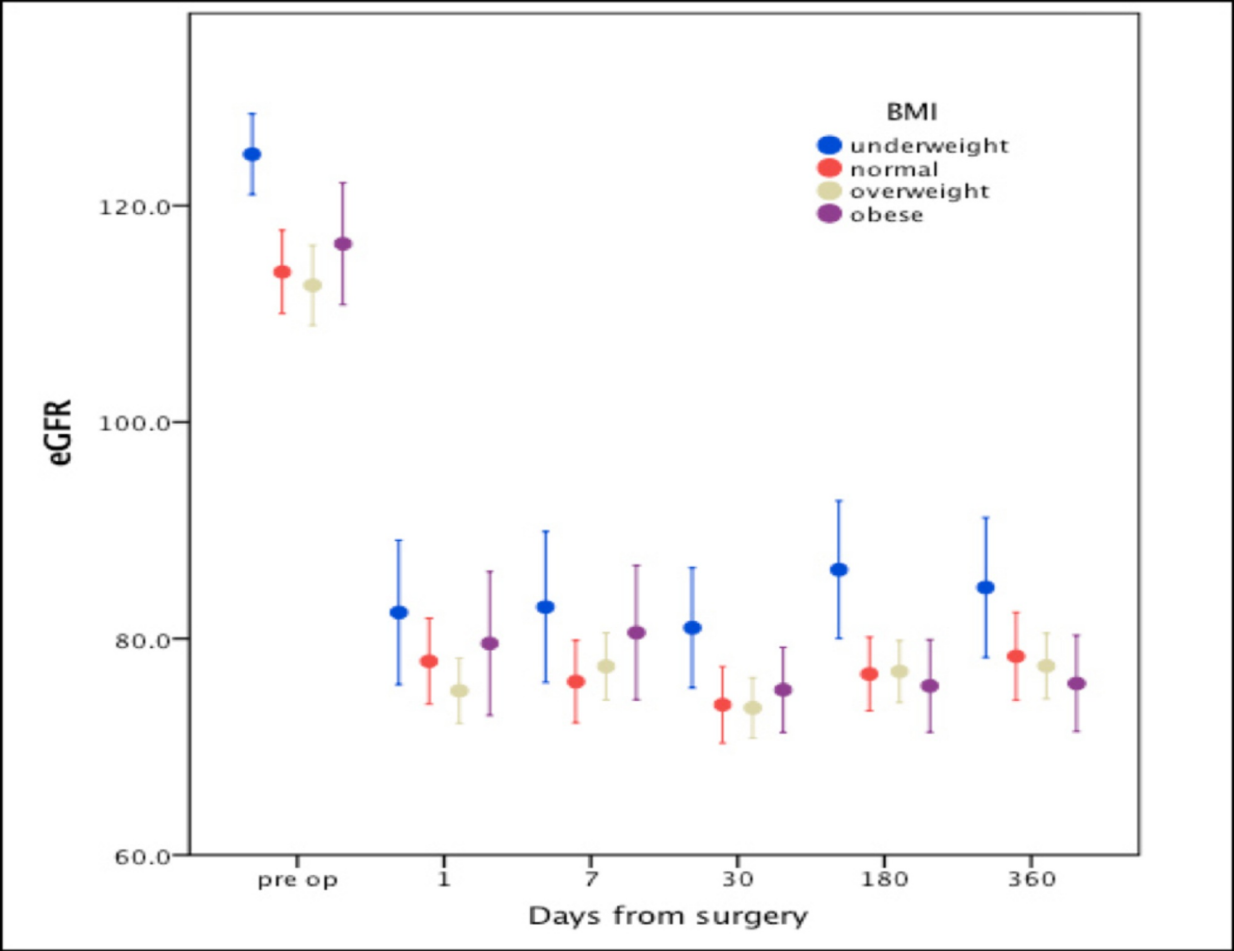
Mean eGFR changes among different BMI groups across time (error bars represent 95% CI) eGFR: estimated glomerular filtration rate; BMI: body mass index

Renal function changes in different experimental sub-groups post-contralateral nephrectomy are illustrated in Figures 1, 2. In spite of a higher eGFR in males (115.1 ±15.6) vs. females (111.3 ±16.8), eGFR dropped more dramatically in male patients post-donation and remained lower after one year (75.2 ±12.3 and 87.7 ±17.2) in males (p: <0.001). Patients aged less than 30 years had a higher eGFR (119.5 ±14.9) compared to patients older than 30 years (109.1 ±15.2) preoperatively, which continued to be higher after one year (82.2 ±15.6 vs. 73.7 ±11.6. p: <0.001). Similarly, non-smokers had better eGFR (115.4 ±16.4) before transplant compared to smokers (112.5 ±15.0), and this trend continued after one year (79.8 ±16.1 vs. 75.1 ±10.9, p: <0.001). eGFR was higher in patients with left nephrectomy compared to those with right nephrectomy preoperatively (115.2 ±15.3 vs. 108.5 ±18.7), and this trend persisted one year after the procedure (78.3 ±14.7 vs. 75.4 ±12.2, p: <0.001). Underweight patients had the best eGFR (124.3 ±5.9), compared to patients who were normal weight (113.8 ±15.6), overweight (112.6 ±17.1), and obese (116.4 ±14.2). Conversely, one-year postoperative eGFR was lower in obese patients (75.8 ±11.2) compared to those who were overweight (77.4 ±14.0), normal weight (78.3 ±16.5), and underweight (84.7 ±9.7). Nevertheless, only underweight patients' eGFR was significantly different from that of other groups.

**Table 3:** Linear regression model for eGFR at the end of the first year eGFR: estimated glomerular filtration rate; BMI: body mass index; MAP: mean arterial pressure; HTN: hypertension

Variables	B	95% CI		P-value
		Lower bound	Upper bound	
Age	-0.315	-0.53	-0.1	0.004
Baseline eGFR	0.332	0.222	0.442	<0.001
Baseline BMI	-0.497	-0.928	-0.067	0.023
MAP	0.088	-0.095	0.27	0.347
Kidney size	3.298	1.432	5.163	0.001
Gender (female)	15.937	11.476	20.399	<0.001
Smoker	0.292	-3.389	3.974	0.876
HTN	-4.301	-26.427	17.826	0.703
Nephrectomy (right)	-0.837	-5.923	4.249	0.747
Arteries >1	-8.429	-24.912	8.055	0.316

Variables predicting eGFR of >70 ml/min/1.73 m^2^ at the end of the first year after contralateral nephrectomy

**Figure 3:**
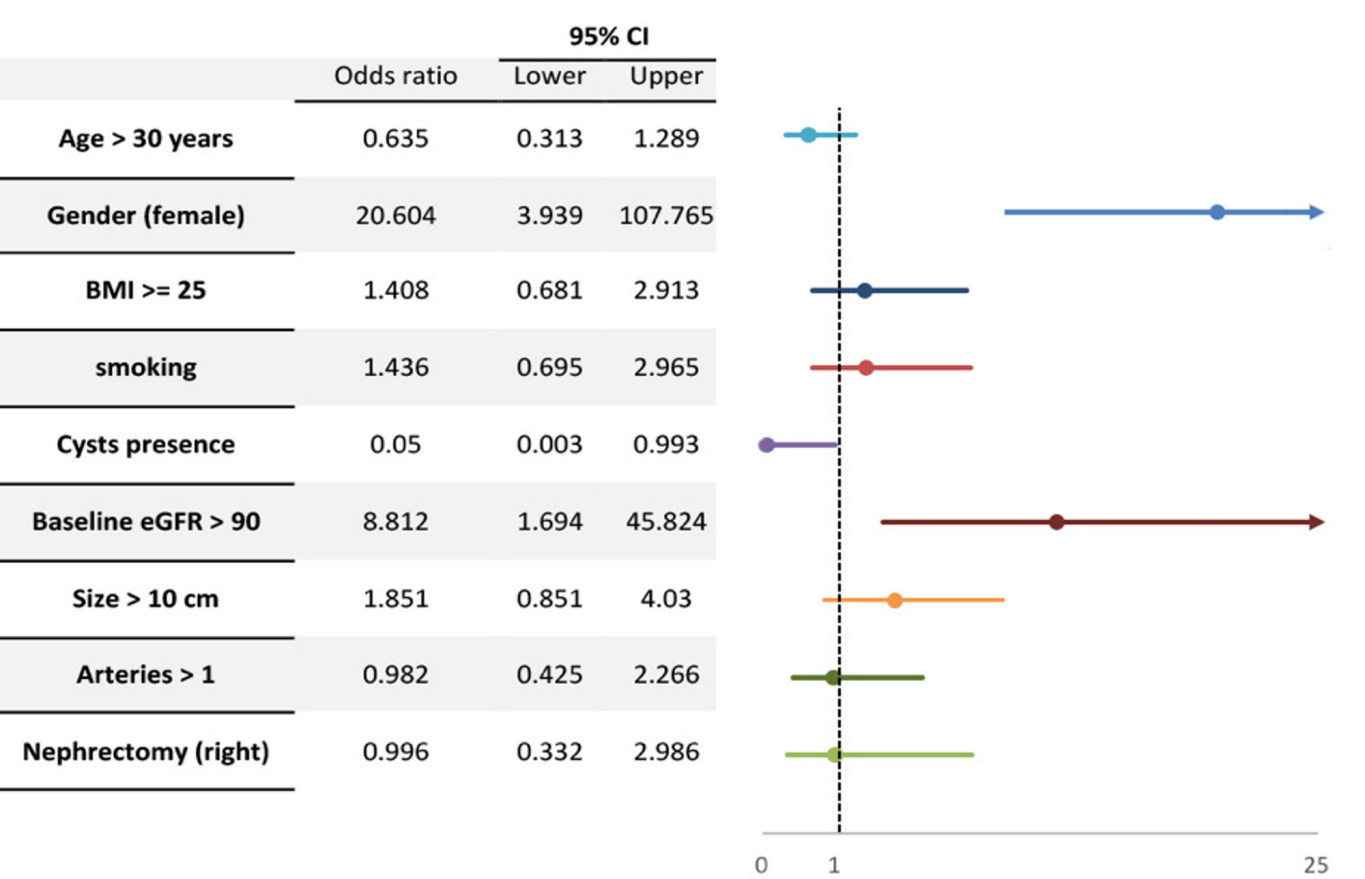
Adjusted odds ratios with 95% confidence intervals for eGFR of more than 70 after one year of surgery BMI: body mass index; eGFR: estimated glomerular filtration rate

Table 4 demonstrates the results of multivariate logistic regression analysis. Independent variables entered into the predictive model include age, gender, BMI, smoking status, presence of renal cysts, baseline eGFR, kidney size, presence of >1 artery, and nephrectomy side. We found that only female gender [odds ratio (OR): 20.6, 95% CI: 3.9-107.7, p: <0.001] and having baseline eGFR of more than 90 (OR: 8.8, 95% CI: 1.6-45.8, p = 0.01) were significant predictors of having an eGFR of more than 70 after one year of the surgery. Figure 3 demonstrates adjusted odds ratios with 95% confidence intervals for eGFR of more than 70 after one year of surgery for the same variables.

**Table 4:** Logistic regression model for the prediction of eGFR of more than 70 at the end of the first year BMI: body mass index; eGFR: estimated glomerular filtration rate

Variables		95% CI		P-value
	Odds ratio	Lower bound	Upper bound	
Age >30 years	0.635	0.313	1.289	0.209
Gender (female)	20.604	3.939	107.765	<0.001
BMI ≥25	1.408	0.681	2.913	0.356
Smoking	1.436	0.695	2.965	0.328
Cyst presence	0.05	0.003	0.993	0.049
Baseline eGFR >90	8.812	1.694	45.824	0.01
Kidney size >10 cm	1.851	0.851	4.03	0.121
Arteries >1	0.982	0.425	2.266	0.965
Nephrectomy (right)	0.996	0.332	2.986	0.994

## Discussion

In this retrospective cross-sectional study of kidney donors, we assessed the changes in the remaining kidney function after contralateral nephrectomy and sought to identify the factors that may predict the degree of kidney function after contralateral nephrectomy. Many previous studies have evaluated the ability of the remaining kidney to achieve a specific value of eGFR post-kidney donation, and an eGFR of greater than 70 ml/min is considered to represent adequate renal compensation. The proportion of donors that reach this value has varied per series, between 10 and 91% [19,20]. Several studies have described how the remaining kidney increases the GFR after nephrectomy of the contralateral kidney. This includes an increase in renal blood flow immediately after nephrectomy and an increase in kidney size (glomerular hypertrophy) after several weeks and these changes lead to an increase in GFR to reach 70% of the previous renal function [11,16,18,21,22].

Our study results are similar to other studies that have assessed the variables affecting kidney function post-contralateral nephrectomy [16,23,24]. It shows that low serum creatinine levels and high eGFR pre-nephrectomy were the main factors that could significantly predict better kidney function at one year after contralateral nephrectomy. Donors of the female gender were also found to have better serum creatinine than those of the male gender after contralateral nephrectomy. This finding may be explained by the lower muscle mass in females. Other factors such as donor age, BMI, smoking status, presence of hypertension, renal cyst, size of the kidney, number of renal arteries, or side of nephrectomy were not significantly associated with the level of the remaining kidney function at one year after contralateral nephrectomy.

In our cohort, the mean eGFR and serum creatinine before kidney donation were 114.31 ±15.94 ml/min/1.73 m^2^ and 71.60 ±10.62 mmol/min, respectively. At the one-year follow-up, the mean eGFR was 77.97 ±14.44 ml/min/1.73 m^2^ and serum creatinine was 100.84 ±20.15 mmol/min. The absolute reduction in eGFR at one year after the donation was 37 ml/min/1.73m^2^ and the absolute rise in creatinine at one year after the donation was 30 mmol/min. We observed no significant difference between immediate post-donation serum creatinine and eGFR values and those at one year after the donation (Table 2). This finding may suggest that renal compensation occurs immediately due to physiological changes causing hyperfiltration rather than hypertrophy that may take a longer time to develop.

Shiraishi et al. have reported that factors such as age, male sex, and BMI correlated strongly with declining renal function [25]. In our study, we investigated the effect of these variables on renal function post-kidney donation (Table 3, Figure 1). We found that eGFR dropped more dramatically in male donors post-donation compared to female donors (75.2 ±12.3 vs. 87.7 ±17.2, p: <0.001), and it remained lower after one year. Aging is known to be associated with glomerulosclerosis, which may reduce renal compensation [26]. We compared donors younger than 30 years with those older than 30 years and found that younger patients had higher eGFR preoperatively (119.5 ±14.9 vs. 109.1 ±15.2) and this trend continued after one year (82.2 ±15.6 vs. 73.7 ±11.6); however, this difference was not statistically significant (p = 0.209).

Regarding smoking, several studies have proven its negative effect on renal function [25,27]. In our study, 77 (40.5%) donors were smokers; non-smokers in our cohort had better eGFR (115.4 ±16.4) before transplant compared to smokers (112.5 ±15.0) and this was true after one year as well (79.8 ±16.1 vs. 75.1 ±10.9), although this difference was not statistically significant (p = 0.876).

Obesity is a well-known cause of kidney disease; it causes hyperfiltration, proteinuria, and glomerulosclerosis. In our study, obese donors had lower eGFR at one year after the donation (75.8 ±11.2) compared to those who were overweight (77.4 ±14.0), normal weight (78.3 ±16.5), and underweight (84.7 ±9.7). However, only underweight patients' eGFR was significantly different from that of other groups (figure 2).

Only a few studies have been conducted so far to identify the factors that predict renal compensation post-kidney donation. To reach a consensus as to which pre-donation renal function parameters are better predictors regarding renal outcomes, multiple factors have been investigated, such as donor age, gender, BMI, hypertension, and smoking. However, it has turned out that none of these are good predictors for renal outcomes after kidney donation. In addition, there are no existing studies that correlate between the degree of renal compensation and long-term renal outcomes. Hence, more studies are required with longer follow-up duration to analyze factors predicting renal compensation and to identify whether the renal compensation rate predicts the long-term renal function.

Our study has several limitations. Primarily, it was a retrospective observational study conducted in a single center with a limited number of donors who belonged to the same ethnicity; we also had a low number of donors who were above 40 years old and a low number of donors with hypertension. Moreover, we relied on a single method for renal function estimation, and our follow-up duration was short.

## Conclusions

Based on our findings, female gender and pre-donation low serum creatinine and high eGFR values were the main factors that significantly predicted better kidney functions at one year after contralateral nephrectomy. However, further studies with longer follow-up periods are needed to better assess the factors that predict renal compensation and the renal compensation rate's suitability as a prognostic measure for long-term renal outcomes.
